# A global compendium of human Crimean-Congo haemorrhagic fever virus occurrence

**DOI:** 10.1038/sdata.2015.16

**Published:** 2015-04-14

**Authors:** Jane P Messina, David M Pigott, Kirsten A Duda, John S Brownstein, Monica F Myers, Dylan B George, Simon I Hay

**Affiliations:** 1 Spatial Ecology and Epidemiology Group, Department of Zoology, University of Oxford, South Parks Road, Oxford OX1 3PS, UK; 2 Department of Pediatrics, Harvard Medical School and Children's Hospital Informatics Program, Boston Children’s Hospital, Boston, Massachusetts 02115, USA; 3 Fogarty International Center, National Institutes of Health, Bethesda, Maryland 20892, USA

**Keywords:** Viral infection, Literature mining, Epidemiology, Viral epidemiology

## Abstract

In order to map global disease risk, a geographic database of human Crimean-Congo haemorrhagic fever virus (CCHFV) occurrence was produced by surveying peer-reviewed literature and case reports, as well as informal online sources. Here we present this database, comprising occurrence data linked to geographic point or polygon locations dating from 1953 to 2013. We fully describe all data collection, geo-positioning, database management and quality-control procedures. This is the most comprehensive database of confirmed CCHF occurrence in humans to-date, containing 1,721 geo-positioned occurrences in total.

## Background & Summary

The quality of reporting of Crimean-Congo haemorrhagic fever virus (CCHFV) infection is inconsistent by country and by region and is often biased by difficulties in diagnosis, limited resources for diagnostic testing and the variable reporting capacities of national health systems. Furthermore, active surveillance of human CCHFV infection is rare, so it is difficult to gauge the limits and intensity of transmission in a consistent manner across the world. As such, this database focuses on known occurrences rather than incidence of human CCHFV infection, with each occurrence identified by its unique geographical location on the globe and the year in which it occurred between 1953 and 2013. Sources of information include published literature, case reports and informal online sources, described in detail in our methods section.

Because the purpose of this database is to enable the identification of all locations of known occurrences of human CCHFV infection globally, many different types of information sources were used for compiling the database. These included individual case reports, reports of many cases in a particular location over a given period of time (up to several years), and active surveillance studies. As such, no information about the number of cases represented by each occurrence record was recorded, as the denominator would not be consistent over space or time, and as such attempts to model incidence or prevalence of CCHF would be a misuse of the database. Rather, with this database it is possible to model the probability of occurrence of CCHFV transmission to humans with a high degree of spatial resolution, for example as Bhatt *et al.*^1^ did for dengue. In that study, the global probability of occurrence of human dengue infection was derived over long-term average conditions at a 5 km×5 km resolution using a similar occurrence database as we describe here for CCHF, as well as a suite of environmental covariates. It would be unnecessary to repeat this type of labour-intensive data collection for these diseases or others for which this work has already been done^2,3^. In drawing upon information from studies in multiple locations, this type of database is intended to allow inferences to be made about the likelihood of disease transmission in locations with little or no information and thereby inform future research and surveillance efforts. Because the locations of disease occurrence are recorded at the finest level of detail possible (i.e., points when the exact location was known or else polygons for administrative units when this was the best information available), this database allows these environmental characteristics to be determined as accurately as possible in a consistent manner at larger spatial scales.

It should be noted that we did not discriminate studies or reports based upon the clinical outcome of the human CCHF infections they reported; this information was not consistently reported across all of the varied sources and we aimed to be comprehensive in our inclusion of all known locations of confirmed human CCHFV infection. We did, however, exclude cases where a healthy individual tested positive for CCHFV antibodies due to potential cross-reactivity with other viruses and the inability to determine the site and time of infection.

We describe all data collection processes in full, as well as the geo-positioning, quality-control and database-management procedures. The database’s construction from many different sources of information makes it the best currently available standardised data available on global CCHFV infection in humans, comprising 1,721 geo-positioned occurrences in total worldwide. The database is not necessarily confined to use for global analyses, as it contains enough detailed information for certain parts of the world to carry out modelling at a regional or even sub-national scale. Regions and countries are specified in addition to the specific location of every record in order to facilitate sub-setting of the database for the user’s needs.

## Methods

### Data collection

An occurrence database comprising point (e.g., town or city) or polygon (e.g., county or province) locations of confirmed CCHF infection presence was compiled from peer-reviewed literature, Genbank records, and HealthMap alerts. A literature search was conducted on PubMed and Web of Science using the terms ‘CCHF’ or ‘Crimean Congo Hemorrhagic Fever’ or ‘Crimean Hemorrhagic Fever’ or ‘Congo Hemorrhagic Fever’. The same terms were used in our Genbank search. An occurrence was defined as one or more laboratory or clinically confirmed infection(s) with CCHFV occurring at a unique location (the same polygon, or 5 km×5 km pixel for points) within one calendar year. All occurrence data underwent manual review and quality control to ensure reliability and precision of geo-positioning. Original data sources can be provided in PDF format by requesting the files from the corresponding author.

Informal online data sources were collated automatically by the web-based system HealthMap (http://healthmap.org) as described elsewhere^4^. Briefly, HealthMap is an online infectious disease outbreak-monitoring system that captures data from a range of electronic sources in nine different languages. The system performs hourly scans of online news aggregators, listservs, electronic disease surveillance networks and public health outbreak report feeds. It captures four fields: headline (the headline, title or subject line), date (publication date), description (a brief summary) and information text (the main content of the article or report). The information text is passed to HealthMap’s classification engine, which parses out one or more disease names and outbreak locations using dictionaries of disease and location patterns. The system then uses a separate algorithm to assign relevance scores that classify alerts as (i) breaking (information about a new outbreak or new information about an on-going outbreak), (ii) context (content about research, policy or background on a particular disease), (iii) warning (articles that warn about the potential for an outbreak), (iv) not disease-related (articles that are captured by the system because they contain words that match disease names in the dictionary but are not in fact about an infectious disease) or (v) old news (an article that mentions a historical outbreak). Finally, HealthMap handles duplicates by aggregating together highly similar alerts such as those released by a news wire service and published in multiple periodicals. The requirements for including a CCHF occurrence record from the HealthMap data set in our database were that the article or report contained one of thirty-six key words or phrases in twenty different languages, including ‘CCHF’, ‘CCHFV’, and ‘Crimean-Congo’ in English. The article must also have been classified by the system as ‘breaking’. The HealthMap data set included in the current database was last updated on 26th May 2012.

### Geo-positioning of data

All available location information was extracted from each peer-reviewed article and PROMED case report. The site name was used together with all contextual information provided about the site position to determine its latitudinal and longitudinal coordinates using Google Maps (https://www.maps.google.co.uk/). Place names are often duplicated within a country, so the contextual information was used to ensure the correct site was selected. When the site name was not found, the contextual information was used to scan sites in the approximate area to check for names that had been transliterated in Google Maps in a different way to the published article (e.g., Imichli and Imishly). If the study site could be geo-positioned to a specific city, town or village, its centre was recorded and termed a ‘point location’ (i.e., associated with a specific latitude and longitude). Point occurrences also included explicit co-ordinates supplied in the article. In reports where more accurate details about the specific location within the city, town or village were available (such as a specific suburb), this was used to define the point occurrence. If the study site could only be identified at an administrative area level (e.g., province or district), the latitude and longitude of a point within the area, along with its name and administrative level, was recorded from Google Maps and then later overlaid in a geographic information system (GIS) to identify the appropriate polygon for the administrative unit. All administrative units were as recognised by the FAO Global Administrative Unit Layer (GAUL) system^3^. These occurrences referring to an area were termed ‘polygon locations’. Reports of autochthonous (locally transmitted) cases or outbreaks were entered as an occurrence within the country in which transmission occurred. If imported cases were reported with information about the site of infection, they were geo-positioned to the country where transmission occurred. If imported cases were reported with no information about the site of contagion, they were not entered into the database. All formal occurrence records underwent spatial and temporal standardisation as described below, in order to ensure consistent definition of an occurrence before undergoing technical validation.

Geo-positions for the HealthMap data were generated using a custom-built gazetteer, or geographic dictionary, of over 4,000 relevant phrases and place names and their corresponding geographic coordinates. The system uses a look-up tree algorithm that searches for matches between sequences of words in alert info text and sequences of words in the gazetteer. When a match is found, a set of rules are applied which attempt to determine the relevance of the place name to the outbreak that is being reported based on the position of the phrase in the report text. The gazetteer includes place names at a range of spatial resolutions (e.g., neighbourhoods, cities, provinces and countries) and uses certain phrases to trigger exclusion of a place name (e.g., Brazil nut). All HealthMap records were added to the unstandardised database and then underwent spatial and temporal standardisation, as well as technical validation, along with the records from the literature and ProMED reports.

### Occurrence database management locational and temporal standardisation

As the database was compiled from many different sources and by multiple persons, it was first necessary to standardise the data entries such that identical locations which may have been geo-positioned slightly differently were given the same unique identifier. To do this, polygon records were all assigned a unique administrative code by overlaying the recorded geographic coordinates in the GIS with corresponding administrative unit shapefiles^5^. Point records were given the same unique identifier if they lay in the same 5 km×5 km pixel within a global grid. Finally, any record associated with a polygon measuring larger than 1°×1° at the equator was removed from the database, although the authors are happy to provide the records for these polygons upon request.

It was next necessary to temporally standardise the database, as the collected CCHF occurrence data came in a variety of temporal forms. For example, some sources reported multiple cases in a single location throughout a year with no finer-scale temporal information. However, in other sources (particularly online sources), multiple cases in the same location throughout the year were presented as a new report each time subsequent transmission occurred. Furthermore, many sources described endemic transmission occurring across multiple years. As a result, we chose to define a single occurrence at a given unique location (as identified above) as one or more confirmed human cases of CCHFV infection occurring within one calendar year in that location, as this was the finest temporal resolution available across all records. This involved a procedure which: (i) disaggregated any records which were in the same location but spanning multiple years into individual records for each respective year; and then (ii) aggregated all records with the same unique location identifier and occurring within the same year to form a single occurrence record. It should be emphasized that because the studies and case reports from which data was compiled represent various types of both active and passive surveillance, the resulting database alone cannot reveal the actual frequency of CCHF occurrences, and that each unique location where any CCHFV infections occurred were only assigned one occurrence per year, regardless of the number of infections reported. The database next and finally underwent technical validation, as described below.

## Data Records

**Figure 1:**
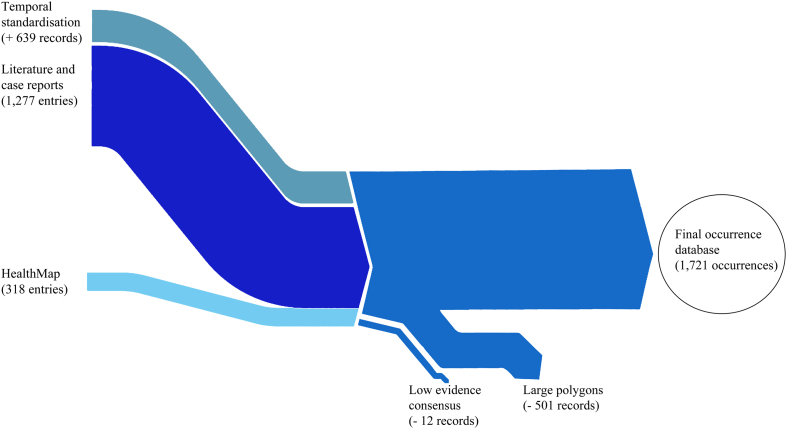
Occurrence data processing summary, beginning with the raw inputs and showing the proportion of data gained or lost through the stages of temporal standardisation and quality control before reaching the final occurrence database.

**Figure 2:**
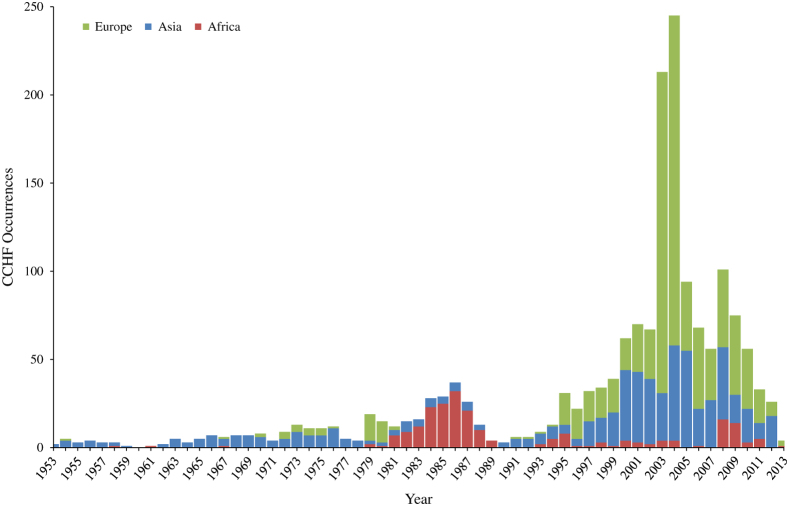
The numbers of occurrence locations per year separated by region.

The database is publicly available online (Data Citation 1) as a comma-delimited file for ease of use and the ability to import it into a variety of software programs. Each of the 1,721 rows represents a single occurrence record (one or more CCHF cases in the same unique location within a single calendar year). A summary of the data management procedure, beginning with the raw inputs and showing the proportion of data lost through the stages of quality control before reaching the final occurrence database, is provided in Fig. 1. Only three records were entered at the country level, and were all in countries with an area greater than 1 degree squared at the equator, so no country-level records existed in the final database as can be seen below. Fig. 2 displays the numbers of occurrence locations per year separated by region. The fields contained in the database are as follows:

1. **OCCURRENCE_ID:** Unique identifier for each occurrence in the database after temporal and locational standardisation.
2. **LOCATION_TYPE:** Whether the record represents a point or a polygon location.
3. **ADMIN_LEVEL:** The administrative level which the record represents when the location type is a polygon. Values are 1 (state or province), 2 (district) and −999 when the location type is a point.
4. **GAUL_AD1:** The first-level GAUL code which identifies the Admin-1 level occurrences as well as the Admin-1 polygon within which any smaller polygons and points lie.
5. **GAUL_AD2:** The second-level GAUL code which identifies the Admin-2 level occurrences as well as the Admin-2 polygon within which any points lie. Values of −999 are assigned when the polygon was Admin-1 level.
6. **UNIQUE_LOCATION:** A unique identifier created for all locations (both points and polygons) based upon the unique point locations (in same 5 km×5 km pixels) and the GAUL codes.
7. **X:** The longitudinal coordinate of the point or polygon centroid (WGS1984 Datum).
8. **Y:** The latitudinal coordinate of the point or polygon centroid (WGS1984 Datum).
9. **YEAR:** The year of the occurrence.
10. **COUNTRY:** The name of the country within which the occurrence lies.
11. **REGION:** The region within which the occurrence lies—values are Asia, Europe, and Africa (includes the Arabian peninsula).

## Technical Validation

The following procedures were carried out on the final database to ensure the accuracy and validity of the occurrence records.

1. A raster distinguishing land from water was created at a 5 km×5 km resolution and was used to ensure all disease occurrences were positioned on a valid land pixel.
2. We cross-validated all of the unique occurrence locations against CCHFV transmission extent based upon an evidence-based consensus score, derived in a similar manner as Brady *et al.*^6^ previously carried out for dengue. In brief, this classification was determined according to a qualitative evidence base that assessed consensus among a wide variety of evidence types on presence or absence of human CCHF cases at a national and sometimes sub-national level. This consensus ranged from complete agreement on absence (score of −100) to complete agreement on presence (100). We chose to exclude points in areas with scores of less than −25. This conservative criterion was intended to preserve points in areas of uncertainty on CCHF status.
3. A random sub-sample of HealthMap occurrence points were manually checked to identify common geo-positioning problems which were rectified in the final database.

The result is a database consisting of 1,721 geo-positioned occurrences in total worldwide, broken down by region and location type in Table 1.

In addition to the main comma-delimited file, three Supplementary Files are included as part of the file set which can be found online (Data Citation 1). These include two text documents: (i) a bibliography of the published literature used for inputting occurrence records, and (ii) a list of accession ID numbers for those records obtained from GenBank. The third Supplementary File is an additional comma-delimited file containing the HealthMap records entered into the database (before standardization and technical validation). This database contains information on the year of each report, as well as the latitude and longitude of the disease occurrence and the source from which this information was extracted.

## Additional information

**How to cite this article:** Messina, J. P. *et al.* A global compendium of human Crimean-Congo haemorrhagic fever virus occurrence. *Sci. Data* 2:150016 doi: 10.1038/sdata.2015.16 (2015).

## Supplementary Material



## Figures and Tables

**Table 1:** Occurrences broken down by region and location type.

**Location Type**	**Africa**	**Asia**	**Europe**	**Total**
point	184	740	473	1397
polygon	43	150	131	324
Total	227	890	604	1721
